# Polymorphism in the PBX1 gene is related to cystinuria in Brazilian families

**DOI:** 10.1111/jcmm.13981

**Published:** 2018-11-18

**Authors:** Sabrina T. Reis, Katia R. M. Leite, Giovanni S. Marchini, Ronaldo M. Guimarães, Nayara I. Viana, Ruan C. A. Pimenta, Fabio C. Torricelli, Alexandre Danilovic, Fábio Carvalho Vicentini, William Carlos Nahas, Miguel Srougi, Eduardo Mazzucchi

**Affiliations:** ^1^ Laboratory of Medical Investigation (LIM55) Division of Urology University of Sao Paulo Medical School Sao Paulo Brazil; ^2^ Endourology and Stone Disease Section Division of Urology University of Sao Paulo Medical School Sao Paulo Brazil

**Keywords:** cystine, genetics, SNP

## Abstract

The aim of our study was to determine regions of loss of heterozygosity, copy number variation analysis, and single nucleotide polymorphisms (SNPs) in Brazilian patients with cystinuria. A linkage study was performed using DNA samples from six patients with cystinuria and six healthy individuals. Genotyping was done with the Genome‐Wide Human SNP 6.0 arrays (Affymetrix, Inc., Santa Clara, CA, USA). For validation, SNPs were genotyped using a TaqMan^®^ SNP Genotyping Assay Kit. The homozygote polymorphic genotype of SNP rs17383719 in the gene PBX1 was more frequent (*P* = 0.015) in cystinuric patients. The presence of the polymorphic allele for this SNP increased the chance of cystinuria by 3.0‐fold (*P* = 0.036). Pre‐B‐cell leukaemia transcription factor 1 (PBX1) was overexpressed 3.3‐fold in patients with cystinuria. However, when we compared the gene expression findings with the genotyping, patients with a polymorphic homozygote genotype had underexpression of PBX1, while patients with a heterozygote or wild‐type homozygote genotype had overexpression of PBX1. There is a 3‐fold increase in the risk of the development of cystinuria among individuals with this particular SNP in the *PBX1* gene. We postulate that the presence of this SNP alters the expression of PBX1, thus affecting the renal absorption of cystine and other amino acids, predisposing to nephrolithiasis.

## 1 INTRODUCTION

Cystinuria is an autosomal recessive disorder characterized by impaired transport of cystine and dibasic amino acids (lysine, ornithine and arginine) in the proximal renal tubule and gastrointestinal tract. The prevalence is not exactly known, though it is estimated to affect one in every 7000 neonates.^1^ Cystinuria is a cause of recurrent kidney stone formation and accounts for approximately 3% of nephrolithiasis cases in humans. Treatment includes increased water consumption, urine alkalization and specific medications that target the cystine molecule. When the stone burden is significant, the stones must be treated by interventional surgical practices.^2^

Although there have been just a few advances in the prevention and treatment of cystinuria in recent years, several promising genetic studies reinforce the hope for better care of affected patients. So far, two genes have been implicated in the genesis of cystinuria: Solute Carrier Family 3 Member 1 (*SLC3A1*) and Solute Carrier Family 7 Member 9 (*SLC7A9*). However, more recent studies have not identified mutations in *SLC3A1* or *SLC7A9* in all patients with this disease, and the study results differ according to the geographical location.^3^

This study describes novel variants and consists of the molecular characterization of cystinuria in Brazilian families. In this investigation, genomic aberrations associated with cystinuria were examined using genome‐wide human single nucleotide polymorphism (SNP) arrays. This approach has the advantages of SNP genotyping and allows the detection of copy number variation (CNV) breakpoints and loss of heterozygosity (LOH), used for surveying segments for allelic losses. Here, we found an association between a SNP in Pre‐B‐cell leukaemia transcription factor 1 (*PBX1*) gene and the risk of development of cystinuria.

## 2 MATERIALS AND METHODS

### 2.1 Patients

This study included a sample of 18 patients under treatment by a tertiary academic care centre. Eight patients with cystine stones (mean age 27 years, four men and 4 women) and 10 controls (mean age 39 years, three men and seven women) without the evidence of any calculi on urinary tract ultrasound or a previous history of the disease were included in the analysis. Clinical diagnosis of cystinuria was based on the biochemical measurement of cystine in the urine and the presence of cystine renal stones. The serum and urinary metabolic profile of all patients were evaluated by NM‐BAPTA method.

For the microarray study, 12 patients out of 18 were selected and divided into two groups: patients with cystine stones (n = 6) and patients without stones and no familial history of cystinuria (n = 6). To validate the results of the genetic polymorphisms by qPCR, we genotyped all 18 patients, 10 without stones and 8 patients with cystinuria.

Patients in both groups provided written informed consent to participate in the study and allowed their biological samples to be genetically analysed. Approval for the study was given by the Institutional Board of Ethics (CAPPesq: Comissão de Ética para Análise de Projetos de Pesquisa) under number 898.995.

### 2.2 Affymetrix SNP 6.0 analysis

The whole blood was collected from patients using vacutainer system. Genomic DNA was isolated using a QiaAmp DNA Mini Kit (Qiagen, CA). Total genomic DNA (250 ng) was digested with a restriction enzyme (*Nsp*I or *Sty*I) and ligated to adaptors that recognized the cohesive four base pair (bp) overhangs. All fragments resulting from restriction enzyme digestion were substrates for adaptor ligation. A generic primer that recognizes the adaptor sequence was used to amplify adaptor‐ligated DNA fragments. PCR conditions were optimized to preferentially amplify fragments in the 200‐1100 bp size range. The amplified DNA was then fragmented, labelled, and hybridized to Genome‐Wide Human SNP 6.0 arrays (Affymetrix, Inc.), this array including more than 906 600 SNPs and more than 946 000 probes for the detection of CNV. After 16 hours of hybridization at 49°C, the arrays were washed by a Fluidics Station 450 and scanned by a Gene Chip Scanner 3000 (Thermo Fisher Scientific, Waltham, MA, USA).

### 2.3 SNP validation by qPCR

Seven SNPs were genotyped using a TaqMan^®^ SNP Genotyping Assay Kit and an ABI 7500 fast system (Applied Biosystems, Foster City, CA, USA). SNP‐specific polymerase chain reaction (ss‐PCR) primers and fluorogenic probes were designed using Primer Express (Applied Biosystems) (Table S1). The fluorogenic probes were labelled with a reporter dye (FAM or VIC) and were specific for one of the two possible bases identified for that site in the gene sequence.

The target sequence was amplified in a 10 μL reaction volume that contained 5 μL of TaqMan^®^ Universal PCR Master Mix, 0.25 μL of SNP Genotyping Assay (primers and probes, Table S1), 1 μL of genomic DNA, and 3.75 μL of DNase‐free water. The PCR cycling conditions were 2 minutes at 50°C and 10 minutes at 95°C, followed by 40 cycles of 15 seconds at 95°C and 60 seconds at 60°C.

### 2.4 Real‐time PCR for PBX1 gene expression

We used a mirVana kit (Ambion, Austin, TX, USA) for RNA extraction from whole blood, and cDNA was obtained using a High‐Capacity cDNA Reverse Transcription Kit for RNA according to the manufacturer's recommendations. The PCR reaction for obtaining cDNA was performed with Veriti equipment (Applied Biosystems) according to the following parameters: 10 minutes at 25°C, 120 minutes at 37°C and 5 minutes at 85°C.

The RNA expression levels were analysed by qRT‐PCR using an ABI 7500 Fast Real‐Time PCR System (Applied Biosystems). The target sequences were amplified in a 10 μL reaction containing 5 μL of TaqMan Universal PCR Master Mix, 0.5 μL of TaqMan Gene Expression Assays (Pre‐B‐cell leukaemia transcription factor 1 ‐ *PBX1*: HS00231228_m1), 1 μL of cDNA, and 3.5 μL of DNase‐free water. The PCR cycling conditions were 2 minutes at 50°C, 10 minutes at 95°C, and 40 cycles of 15 seconds at 95°C and 1 min at 60°C. All reactions were performed in duplicate, and TaqMan *B2M* (HS00187842_m1) was utilized as housekeeping gene or endogenous control for gene expression.

### 2.5 Statistical analysis

The statistical analysis of the microarray slides was performed with plink software (http://pngu.mgh.harvard.edu/~purcell/plink). Categorical variables are expressed as numbers and percentages. The associations between genotype and allelic frequencies in the cases and controls were examined by chi‐squared tests and for allelic distribution, the Fisher exact odds ratio (OR) and the corresponding 95% confidence intervals (CIs) were calculated. To compare the urine and serum metabolic profile between cases and controls, we used Student's *t* test. Statistical analysis was performed with SPSS 19.0 (Chicago, IL, USA) for Windows, and significance was set at *P* ≤ 0.05.

For gene expression we used the ∆∆CT method to calculate the relative expression of *PBX1* using the formula ∆∆CT. The fold change in gene expression was calculated as 2^−∆∆CT^.

## 3 RESULTS

### 3.1 Serum and urinary analysis

The mean age of the cystinuric patients and control group cases was 28.5 ± 10.9 and 39.2 ± 15.9 years, respectively (*P* = 0.126). The serum and urinary metabolic profile of all patients are shown in Tables S2 and S3, respectively. Seven patients with cystinuria were taking citrate at the time of urine collection. The only metabolic difference between the groups was a higher level of serum uric acid in cystinuric patients (6.07 ± 1.88 vs 4.60 ± 0.96 mg/dL; *P* = 0.047).

### 3.2 Copy number variation analysis

For the CNV, no statistically significant comparison could be attained between cases and controls due to our small sample size. Nevertheless, the descriptive analysis depicted in Table S4 highlights that the changes found were not specific to either cystinuric patients or controls.

### 3.3 Loss of heterozygosity analysis

We examined the distribution of LOH on each chromosome. We found 36 changes in patients with cystinuria and only six variations in the control group. When assessing regions with more frequent LOH in cystinuria patients (Table S5), the changes occurred in one or more regions of SLC genes in 83.3% of patients.

### 3.4 Single nucleotide polymorphism analysis

To confirm the microarray results, qPCR analysis was done for five SNPs associated with the disease in our cohort and in two other cases that have been reported in the literature (Table S1).

The results of the genotyping are shown in Table 1. There was a statistically significant difference in the distribution of the genotypes between patients and controls for the SNP rs17383719. The frequencies of the CC, CT, and TT genotypes were 70.0%, 30.0%, and 0.0% in controls and 14.3%, 28.6% and 57.1% in cystinuric patients, respectively (*P* = 0.015). There were also statistically significant differences in the allelic frequencies between cystinuric patients and controls for this SNP. The C and T alleles were detected in 70.0% and 30.0% of controls and in 14.3% and 85.7% of cystinuric patients, respectively (OR: 2.85; 95% CI: 1.05‐7.071; *P* = 0.036).

**Table 1:** Frequency of genotypes and alleles for the SNPs in the PBX1, SLC7A9, SLC3A1, EXT2, SVIL and PDLIM1 genes in patients with cystinuria and controls

ID SNP	Genotype	Cystinuria (n)	Control (n)	*P*	Allele	Cystinuria	Control	OR	*P*
rs17383719									
PBX1	CC*	14.3% (1)	70.0% (7)	0.015	C*	14.3% (1)	85.7% (6)	1	**0.036**
	CT	28.6% (2)	30.0% (3)		T	70.0% (7)	30.0% (3)	2.85 (1.05‐7.71)	
	TT	57.1% (4)	0.0%						
rs140134166									
SLC7A9	CC*	100.0% (7)	100.0% (10)	NA	C*	100.0% (7)	100.0% (10)	‐	NA
	CT	0.0%	0.0%		T	0.0%	0.0%	‐	
	TT	0.0%	0.0%						
Rs200248046									
SLC3A1	GG*	28.6% (2)	0.0%	0.200	G*	28.6% (2)	0.0%	‐	0.200
	GC	71.4% (5)	100.0% (8)		C	71.4% (5)	100.0% (8)	‐	
	CC	0.0%							
Rs3923808									
EXT2	AA*	71.4% (5)	37.5% (3)	0.091	A*	71.4% (5)	37.5% (3)	1	0.315
	AG	0.0%	50.0% (4)		G	28.6% (2)	62.5% (5)	0.45 (0.12‐1.65)	
	GG	28.6% (2)	12.5% (1)						
Rs913034									
SVIL	AA*	85.7% (6)	10.0% (1)	0.005	A*	85.7% (6)	10.0% (1)	1	**0.004**
	AG	14.3% (1)	20.0% (2)		G	14.3% (1)	90.0% (9)	0.15 (0.02‐0.98)	
	GG	0.0%	70.0% (7)						
Rs7096453									
SVIL	TT*	100.0% (4)	0.0%	0.008	T*	100.0% (4)	0.0%	‐	**0.008**
	TC	0.0%	0.0%		C	0.0%	100.0% (5)	‐	
	CC	0.0%	100.0% (5)						
Rs11188246									
PDLIM1	GG*	14.3% (1)	37.5% (3)	0.569	G*	14.3% (1)	37.5% (3)	1	0.569
	GA	85.7% (6)	62.5% (5)		A	85.7% (6)	62.5% (5)	1.37 (0.74‐2.53)	
	AA	0.0%	0.0%						

Bold values represent P‐value that were significantly different.

*Represents wild‐type genotype or allele.

We also found statistically significant differences for the SNPs rs913034 and rs7096453 but only for allelic frequencies. For the SNP rs913034, the frequency of the polymorphic allele in patients with cystinuria was 10.0%, and in the control group, it was 90.0% (OR: 0.15; 95% CI: 0.02‐0.098; *P* = 0.004). The same pattern occurred for the SNP rs7096453, where the polymorphic allele was present in 100% of cases in the control group and not present in any cystinuric patients (*P* = 0.008).

For SNPs in the *SLC7A9* (rs140134166) and *SLC3A1* (rs200248046) genes, no statistical association was observed between cases and controls. Importantly, no case or control patient had the polymorphic allele for SNP in *SLC7A9*.

### 3.5 *PBX1* gene expression

**Figure 1:**
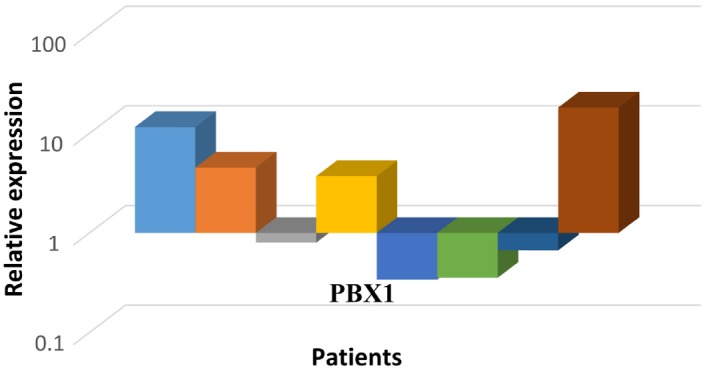
Expression profile of PBX1 in cystinuric patients compared to the control group

Due to the significant results found when we evaluated the SNP in *PBX1*, we evaluated the levels of gene expression of PBX1 by qRT‐PCR (Figure 1). Although *PBX1* was overexpressed by 3.3‐fold in patients with cystinuria compared to the control group, when we compared the gene expression findings with the genotyping, patients with the polymorphic homozygote genotype had underexpression of *PBX1*, while patients with heterozygote or wild‐type homozygote genotypes showed overexpression of *PBX1*.

## 4 DISCUSSION

The markers that have arisen for studies of association and linkage are SNPs, the most common and powerful markers due to their abundance and stability.^4^ There are more than 10 million SNPs catalogued so far with an allelic frequency of at least 5%.^5^ To the best of our knowledge, no other study has searched for SNPs in patients with cystinuria. Our study is a pilot study, and although the sample size is too small for a large‐scale analysis, we found few SNPs that were more frequent in cystinuric patients than in patients without kidney stone disease. We have also validated the results through PCR for SNP rs17383719, which is located in the *PBX1* gene on chromosome 1. No changes in this gene had been associated with the cystinuria phenotype so far; however, we found that the presence of just one polymorphic allele increased the risk of developing the disease three‐fold.

*PBX1* is a member of a transcription factor family, and there are no studies associating this gene with nephrolithiasis, although it has been associated with kidney development. The metanephric kidney develops through reciprocal interactions between the metanephric mesenchyme and the epithelium of the ureteric bud. The TALE (three‐amino acid loop extension) homeodomain transcription factor *PBX1* is expressed weakly in the nephrogenic mesenchyme and strongly in the stroma after ureteric bud invasion. Disruption of *PBX1* results in unilateral kidney agenesis along with a variety of other kidney anomalies.^6^

Single nucleotide polymorphisms may influence promoter activity (gene expression), messenger RNA (mRNA) conformation (stability), and subcellular localization of mRNAs and/or proteins. Therefore, they might ultimately lead to the development of particular diseases. Although there are no studies in the literature linking the *PBX1* gene to nephrolithiasis, we postulate that this SNP in the *PBX1* gene may change the expression or the interaction of this gene with others during kidney development and that this may alter the renal absorption capacity of cystine and other amino acids, increasing the possibility of cystinuria development. For this reason, we performed qRT‐PCR for to evaluate PBX1 gene expression in patients with cystinuria, and curiously, we found overexpression of PBX1 in patients with one or no mutated allele and underexpression of PBX1 in patients with two mutated alleles.

Schnabel et al. (2003) have shown the *PBX1* expression patterns during renal development and demonstrated that the loss of *PBX1* leads to the absence or abnormal formation of metanephric kidneys. In addition, they showed that differentiation of the nephrogenic mesenchyme into nephrons is severely reduced in *PBX1* mutants. These findings established a role for *PBX1* in mesenchymal–epithelial inductive signalling and demonstrated that *PBX1* is a critical regulator of mesenchymal function during renal morphogenesis. Here, we postulate that the presence of a polymorphic homozygote genotype can alter the expression of PBX1 and ultimately affect renal absorption of cystine and other amino acids.

The SNPs rs913034 and rs7096453 are polymorphic alleles that were significantly more prevalent in controls, suggesting that these polymorphisms may play a protective role against the development of cystinuria in the studied population. Interestingly, both were located in the *SVIL* gene (supervillin) on the short arm of chromosome 10. These SNPs were not associated with cystinuria or with other types of stone or kidney diseases. Supervillin is a 205‐kDa F‐actin binding protein originally isolated from bovine neutrophils. This protein is tightly associated with both actin filaments and plasma membranes, suggesting that it forms a high‐affinity connection between the actin cytoskeleton and the membrane. Additionally, *SVIL* regulates cell proliferation through the control of p53, a tumour suppressor gene,^7^ and contributes to cell motility, invasion and rapid turnover of membrane vesicles.^8^

Genomic rearrangements that affect DNA sequences are called structural variants and include insertions, deletions, duplications and inversions.^9^ When the length of a structural variant is 1 kb or longer, it is defined as a CNV. Considering our results from microarrays regarding CNVs, we found that the changes encountered were not specific to cases or controls. For LOH, we found 36 changes in patients with cystinuria and only six in the control group. When assessing regions with more frequent LOH in cystinuric patients, in 83.3% of them, the LOH occurred in one or more regions of SLC genes. The SLC human gene series has grown to now include 52 families and 395 transporter genes. Transporters are assigned to a specific family if the encoded protein has an amino acid sequence at least 20% identical to the proteins of other members of that family.^10^ Defects in many of the solute carriers are associated with human diseases. The finding of LOH in regions of SLC genes deserves attention since the molecular cystinuric changes previously identified have all been in genes of this specific family.^9^ Interestingly, when analysing the frequency of SNPs present in the *SLC7A9* (rs140134166) and *SLC3A1* (rs200248046) genes, in our cohort the SNP in *SLC7A9* was genotyped as wild‐type homozygote for all patients. In the SNP *SLC3A1*, we found a predominance of the heterozygote genotype for cystinuria and control patients. Polymorphisms in these two genes are classic alterations described in the literature in patients with cystinuria.^3^, ^11^, ^12^, ^13^ However, we must emphasize that we have analysed only one change in each of these genes and that recent studies have shown that there are several mutations that may be associated with the phenotype of cystinuria. Furthermore, they might differ according to the geographical location of the studied patients.^14^, ^15^

This is the first study to describe LOH and CNVs in patients with cystinuria. LOH was found in regions where the genes of the SLC family usually associated with cystinuria are located. Furthermore, this study describes novel variants, expands the spectrum of cystinuria genetic variations and contributes to our understanding of the molecular basis of cystinuria in Brazilian patients. The risk of development of cystinuria increased three‐fold in association with the SNP in *PBX1* gene, and the presence of this SNP could change the expression of *PBX1*, affecting renal reabsorption of cystine and other amino acids, possibly predisposing to cystinuria development.

## CONFLICT OF INTEREST

The authors confirm that there are no conflicts of interest.

## AUTHOR CONTRIBUTION

NIV, RCAP and RMG performed the research; STR and KRML analyzed the data; STR wrote the paper; STR, KRML and EM designed the research study; GSM, FCT, AD and FCV contributed the essential reagents and tools; WCN, MS and EM supervised the study.
