# Supplementary material for: Polymorphism in the PBX1 gene is related to cystinuria in Brazilian families
Source: J Cell Mol Med. 2018 Nov 18;23(2):1593–7. doi: 10.1111/jcmm.13981 (PMC6349145; doi:10.1111/jcmm.13981)
Supplement: Supplementary file 1 [file JCMM-23-1593-s001.docx]

**S1 Table.** TaqMan® assays used in the genotyping of the SNPs

| **SNP** | **Gene** |
| --- | --- |
| rs17383719 | PBX1 |
| rs11188246 | PDLIM1 |
| rs7096453 | SVIL |
| rs913034 | SVIL |
| rs3923808 | EXT2 |
| rs140134166 | SLC7A9 |
| rs200248046 | SLC3A1 |

**S2 Table.** Baseline serum metabolic evaluation of cystinuric (cases) patients and individuals without stone disease (controls).

|  | **Cases (n = 8)**  Mean ± SD | **Controls (n = 10)**  Mean ± SD | ***p*** |
| --- | --- | --- | --- |
| **Calcium (mg/dL)** | 9.51 ± 0.69 | 9.22 ± 0.48 | *0.306* |
| **Sodium (mEq/L)** | 139.63 ± 2.13 | 140.40 ± 1.64 | *0.567* |
| **Potassium (mEq/L)** | 4.24 ± 0.30 | 4.40 ± 0.43 | *0.397* |
| **Glucose (mg/dL)** | 89.63 ± 14.23 | 81.30 ± 5.20 | *0.104* |
| **Urea (mg/dL)** | 31.00 ± 5.29 | 25.20 ± 6.56 | *0.060* |
| **Creatinine (mg/dL)** | 1.60 ± 1.48 | 0.83 ± 0.25 | *0.126* |
| **Uric Acid (mg/dL)** | 6.07 ± 1.88 | 4.60 ± 0.96 | ***0.047*** |

**S3 Table.** Baseline urinary metabolic evaluation of cystinuric (cases) patients and individuals without stone disease (controls).

|  | **Cases (n = 8)**  Mean ± SD | **Controls (n = 10)**  Mean ± SD | ***p*** |
| --- | --- | --- | --- |
| **Urine pH** | 6.62 ± 0.51 | 5.85 ± 0.81 | ***0.034*** |
| **Volume (ml) (24 h)** | 2.000.00 ± 676.73 | 1.523.50 ± 402.97 | *0.081* |
| **Calcium (mg/vol) (24 h)** | 114.74 ± 31.94 | 151.07 ± 75.69 | *0.224* |
| **Citrate (mg/vol) (24 h)** | 441.23 ± 221.15 | 520.77 ± 204.84 | *0.441* |
| **Oxalate (24 h)** | 40.88 ± 12.87 | 33.50 ± 11.20 | *0.212* |
| **Sodium (mEq/vol) (24 h)** | 158.13 ± 39.97 | 161.30 ± 34.73 | *0.859* |
| **Potassium (mEq/vol) (24 h)** | 42.05 ± 10.34 | 44.80 ± 8.36 | *0.541* |
| **Uric Acid (mg/vol) (24 h)** | 0.57 ± 0.09 | 0.47 ± 0.11 | *0.053* |

**S4 Table.** Copy number variations found in patients with cystinuria (A) and controls (B).

|  | **A – Patients with cystinuria.** | |  |  |
| --- | --- | --- | --- | --- |
| Ch**romosome** | G**ene** | | T**ype** |  |
| **1** | MIR3675, ESPNP, MST1L, CROCC | | Gain |  |
| **7** | ZNF862, ATP6V0E2-AS1, ATP6V0E2 | | Gain |  |
| **8** | CSMD1 | | Gain |  |
| **8** | FAM66B, DEFB109P1B, USP17L1P, USP17L4, ZNF705G, DEFB107B, FAM90A7P | | Gain |  |
| **8** | FAM66A, DEFB109P1, FAM90A25P, FAM86B2, LOC100506990, LOC729732 | | Gain |  |
| **14** | LINC00516, LOC101101776, POTEM, OR11H2, OR4Q3, OR4M1, OR4N2, OR4K2 | | Gain |  |
| **16** | TP53TG3, TP53TG3B, TP53TG3C, SLC6A10P | | Loss |  |
| **8** | DEFB4B, DEFB103B, DEFB103A, SPAG11B, ZNF705B | | Loss |  |
| **16** | HERC2P4, TP53TG3D, LOC390705 | | Loss |  |
| **16** | RNU6-76P | | Loss |  |
| **19** | LOC100289650, PSG10P, PSG1, PSG6, PSG7, PSG11, PSG2, PSG5, PSG4, LOC284344 | | Loss |  |
| **15** | HERC2P3, GOLGA6L6, GOLGA8CP, NBEAP1 | | Gain |  |
| **15** |  | | Gain |  |
| **15** | OR4N3P, REREP3 | | Gain |  |
| **X** | MAGEA8-AS1, MAGEA8, CXorf40B | | Gain |  |
| **15** | HERC2P3, GOLGA6L6, GOLGA8CP, NBEAP1, POTEB, POTEB2, NF1P2, CT60 | | Gain |  |
| **1** |  | | Gain |  |
| **9** | FAM27C, FAM27A, FAM27E2 | | Gain |  |
| **9** | PGM5P2, LOC440896, FOXD4L6, CBWD6, ANKRD20A4, LOC100133920, FOXD4L5 | | Gain |  |
| **14** | LINC00226 | | Gain |  |
| **16** | TP53TG3, TP53TG3B, TP53TG3C, SLC6A10P, LOC390705, RNU6-76P | | Gain |  |
|  | | **B – Controls.** |  | |
| Ch**romosome** | | G**ene** | **Type** | |
| **2** | | LINC00152, MIR4435-1, MIR4435-2 | Loss | |
| **8** | | FAM66B, DEFB109P1B, USP17L1P, USP17L4, ZNF705G, DEFB107B, FAM90A7P | Loss | |
| **7** | | IMMP2L, DOCK4 | Gain | |
| **8** | | FAM66B, USP17L1P, USP17L4, ZNF705G, FAM90A7P, FAM90A10P, SPAG11A | Loss | |
| **8** | | FAM66D, FAM90A2P, FAM86B1, DEFB130, LOC100133267, FAM66A, LOC649352 | Loss | |
| **14** | | OR4Q3, OR4M1, OR4N2, OR4K2, OR4K5, OR4K1 | Gain | |
| **16** | | LOC283914, LOC146481, LOC100130700 | Gain | |
| **17** | | KANSL1, KANSL1-AS1, LRRC37A, ARL17A, ARL17B, NSFP1, LRRC37A2, NSF | Gain | |
| **1** | | MIR3675, NBPF1, CROCCP2, MST1P2, ESPNP, MST1L | Loss | |
| **2** | | GAL3ST2, NEU4, PDCD1, CXXC11, LOC728323 | Loss | |
| **16** | | HERC2P4, TP53TG3D, LOC390705, TP53TG3, TP53TG3B, TP53TG3C | Loss | |
| **16** | | RNU6-76P | Loss | |
| **1** | |  | Loss | |
| **10** | | NPY4R, LINC00842, HNRNPA1P33, ANXA8, ANXA8L1, FAM25G, FAM25C, | Gain | |
| **14** | | LINC00516, LOC101101776, POTEM, OR11H2, OR4Q3, OR4M1, OR4N2, OR4K2 | Gain | |
| **16** | | LOC283914, LOC146481, LOC100130700 | Gain | |
| **X** | | MAGEA8-AS1, MAGEA8, CXorf40B, LINC00894 | Gain | |
| **15** | | HERC2P3, GOLGA6L6, GOLGA8CP, NBEAP1, POTEB, POTEB2, NF1P2, CT60 | Loss | |
| **1** | | MIR3675, NBPF1, CROCCP2, MST1P2, ESPNP, MST1L | Gain | |
| **7** | |  | Gain | |
| **8** | | DEFB4B, DEFB103B, DEFB103A, SPAG11B, ZNF705B | Loss | |
| **16** | | LOC283914, LOC146481, LOC100130700 | Gain | |
| **X** | | MAGEA8-AS1, MAGEA8, CXorf40B, LINC00894 | Gain | |

**S5 Table.** LOH found in patients with cystine stones.

| **Chromosome** | **Genes** |
| --- | --- |
| 5 | MRPS30, HCN1, EMB, PARP8, LOC642366, ISL1 |
| 10 | MTRNR2L7, ZNF248, ZNF33BP1, ZNF25, ZNF33A, ZNF37A, LOC100129055, HSD17B7P2, SEPT7P9, LOC399744 |
| 16 | GPT2, DNAJA2, NETO2, ITFG1, PHKB, ABCC12, ABCC11, MIR548AE2, LONP2, LOC100507577, SIAH1 |
| 2 | SLC35F6, CENPA, SLC4A1AP, MRPL33, RBKS, BRE-A PAIP2B, ZNF638, DYSF, CYP26B1 |
| 3 | CHL1, CNTN6, CNTN4, CNTN4-AS2, IL5RA, TRNT1, CRBN, LRRN1 |
| 4 | ANKRD50, FAT4, MIR2054, INTU, SLC25A31, HSPA4L, PLK4, MFSD8, |
| 8 | SLC7A2, SLC25A37, NKX3-1, NKX2-6, STC1, ADAM28, ADAMDEC1, ADAM7 |
| 11 | SLC5A12, FSLC22A10, SLC22A9, HRASLS5, LGALS12, |
| 12 | SC6A15, MIR5700, TMCC3, MIR492, KRT19P2, NDUFA12, RMST, MIR1251, MIR135A2, LOC643711 |
| 16 | SLC7A5P1, SLC5A2RBL2, AKTIP, RPGRIP1L, FTO, IRX3 |
| 18 | MIR4744, C18orf32, RPL17-C18orf32, MIR1539, RPL17, SNORD58C, SNORD58A, SNORD58B, |
| 20 | MACROD2-AS1, KIF16B, SNRPB2, OTOR, PCSK2, BFSP1, DSTN, RRBP1, BANF2, SNX5 |
| 20 | MMP9, SLC12A5, SLC13A3, TP53RK, SLC2A10, PARD6B, BCAS4, ADNP, DPM1, MOCS3 MIR3194 |
| 21 | MIR155HG, MIR155, LINC00515, MRPL39, JAM2, ATP5J, GABPA, APP, CYYR1, ADAMTS1, ADAMTS5 |
| 2 | LOC100506076, LOC100506123, ANKRD36B, COX5B, ACTR1B, LOC728537 |
| 11 | OR8J3, OR8K5, OR5J2, OR5T2, OR5T3, OR5T1, OR8H1, OR8K3, OR8K1, OR8J1, OR8U8, OR8U1, OR5R1 |
| 15 | LINC00923, ARRDC4, FAM169B, IGF1R, MIR4714, DNM1P46, ADAMTS17, SPATA41 |
| 16 | SLC5A2, SLC6A10P, DNAJA2, NETO2, ITFG1, PHKB, ABCC12, ABCC11, MIR548AE2, LONP2, LOC100507577 |
| 17 | SLC13A5, SLC16A13, SLC16A11, SLC2A4, SLC35G6, TMEM220-AS1, LINC00675, PIRT, SHISA6, DNAH9 |
| 19 | RIP10, SH2D3A, VAV1, EMR1, EMR4P, FLJ25758, MBD3L5, MBD3L4, MBD3L2, MBD3L3, ZNF557, INSR |
| 19 | IFNL3, IFNL4, IFNL2, IFNL1, LRFN1, GMFG, PGLYRP1, IGFL4, IGFL3, IGFL2, DKFZp434J0226, RNU6-66P, IGFL1 |
| 7 | SMKR1, NRF1, RNA5SP244, MIR182, MIR96, MIR183, UBE2H, ZC3HC1, KLHDC10 |
| 9 | LHX3, QSOX2, DKFZP434A062, GPSM1, DNLZ, CARD9, SNAPC4, SDCCAG3, PMPCA, INPP5E, SEC16A |
| 17 | SLC5A10, SLC47A1, SLC47A2, LINC00854, LINC00910 |
| 19 | SLC39A3, |
| 19 | SLC7A9, SLC7A10, |
| Y | GYG2P1, TTTY15, USP9Y, DDX3Y, UTY, TMSB4Y, VCY1B, VCY, NLGN4Y, NLGN4Y-AS1 |
| 3 | SLC6A20,VPRBP, RAD54L2, TEX264, GRM2, IQCF6, IQCF4, IQCF3, IQCF2, IQCF5 |
| 3 | SLC9C1, SLC35A5, ZXDC, UROC1, CHST13, C3orf22, TXNRD3NB, TXNRD3, NUP210P1, CHCHD6, PLXNA1 |
| 8 | SLC7A2, PDGFRL, SLC18A1, SLC39A14 |
| 8 | LINC00293, LOC100287846, KIAA0146, CEBPD, PRKDC, MCM4, UBE2V2 |
| 8 | SLC30A8, , FHHLA1, KCNQ3, HPYR1, LRRC6, TMEM71, PHF20L1, TG, SLA, WISP1, NDRG1, ST3GAL1 |
| 14 | S SLC25A21, MIR4503, SLC25A21-AS1, RPL36AL, MGAT2, NEMF, ARF6, C14orf182, LOC100506499 |
| 14 | MNAT1, TRMT5, SLC38A6, TMEM30B, PRKCH, KCNH5, RHOJ, GPHB5, PPP2R5E |
| 16 | RVPS35, ORC6, MYLK3, C16orf87, GPT2, DNAJA2, NETO2, ITFG1, PHKB, ABCC12 |
| X | ASB12, MTMR8, ZC4H2, ZC3H12B, LAS1L, FRMD8P1, MSN, MIR223, VSIG4, HEPH, EDA2R, AR |
